# Miller General Hospital

**Published:** 1919-07-05

**Authors:** 


### MILLER GENERAL HOSPITAL.

THE urgent need for greater accommodation was the underlying cry of the annual meeting of the Miller General Hospital, held on Monday in the Hall of the Drapers' Company, Lord Queenborough in the chair. Major J. H. Robinson described the in-patients' accommodation as "lamentably short," considering the fact that the number of out-patients—124,402 last year—was more than a third of the number at the London Hospital. At the latter institution were 933 beds, yet at the Miller there were only seventy-six. He urged that to meet the demand this number should be increased to at least 300. At present very large numbers of patients who acutely needed attendance were being turned away. This need was implicitly urged also by Canon R. P. Roseveau, who pointed out that the hospital served a district of about 500,000 inhabitants, who looked to it entirely for in-patient and out-patient treatment.

As regards its financial position, the hospital finds itself faced with a deficit of £186 10s., though this would have been avoided but for the transfer of £474 6s. 8d. to the account of the Convalescent Home at Bexhill. It was, indeed, felt that to have been able to outlast five years of war at all was cause for satisfaction, for the cost per in-patient had increased about 6s. 1d. per week, and per out-patient 2⅜d. per attendance since last year, and the total expenses of the hospital had more than doubled since 1913. There was, however, a feeling of anxiety and misgiving as to the future, for the cost of living showed no signs of decrease, the present taxation of the hospital's supporters forced some of them to reduce their subscriptions, and a general feeling of uncertainty was everywhere observable.

A pleasing increase in the receipts was, however, the employees' subscriptions, which, from £9 2s. 9d. in 1915, had risen last year to £1,734 18s. 2d. It was urged by several speakers that all employees should endeavour to arrange at their various places of work for regular subscriptions to the hospital—a system which had been adopted in various provincial towns with a good deal of success.

The resignation of Mr. W. H. McMullen from the Ophthalmic Department was referred to by Major Richardson as a blow to the hospital. For seventeen years, he said, Mr. McMullen had done hard and slogging work, and his success was due to his patient zeal. They were losing a most devoted member of the staff. The loss of Dr. Scholefield, who for twenty-three years was medical officer of the out-patients' department, was also deeply deplored.

Sir Cyril Jackson paid a warm tribute to the work of the staff during the past year. If any one class of people more than another had been overworked by the war it was doctors, and the honorary services given by the hospital visiting staff were "priceless." He referred to the work of the hospital in attending to the needs of discharged Service men, adding that it was most necessary for them to have a hospital near their home. Otherwise large numbers of men would not trouble to go for even voluntary treatment.

The Right Hon. H. Pike Pease spoke to the wise management of the Miller General Hospital. He was not in favour of the nationalisation of hospitals, which he considered detrimental to their best interests, but he saw that it might be necessary for the State to make some small contribution to some of the smaller institutions. It was one of the glories of our race, he declared, that so many men and women could be found to give voluntary help in such a splendid cause.

